# Construction and Accuracy Assessment of Patient-Specific Biocompatible Drill Template for Cervical Anterior Transpedicular Screw (ATPS) Insertion: An *In Vitro* Study

**DOI:** 10.1371/journal.pone.0053580

**Published:** 2013-01-10

**Authors:** Maoqing Fu, Lijun Lin, Xiangxue Kong, Weidong Zhao, Lei Tang, Jianyi Li, Jun Ouyang

**Affiliations:** 1 Department of Anatomy, Guangdong Provincial Key laboratory of Medical Biomechanics, School of Basic Medicine Science, Southern Medical University, Guangzhou, Guangdong, China; 2 Department of Orthopedics, Zhujiang Hospital, Southern Medical University, Guangzhou, Guangdong, China; Southern Medical University, China

## Abstract

**Background:**

With the properties of three-column fixation and anterior-approach-only procedure, anterior transpedicular screw (ATPS) is ideal for severe multilevel traumatic cervical instabilities. However, the accurate insertion of ATPS remains challenging. Here we constructed a patient-specific biocompatible drill template and evaluated its accuracy in assisting ATPS insertion.

**Methods:**

After ethical approval, 24 formalin-preserved cervical vertebrae (C2–C7) were CT scanned. 3D reconstruction models of cervical vertebra were obtained with 2-mm-diameter virtual pin tracts at the central pedicles. The 3D models were used for rapid prototyping (RP) printing. A 2-mm-diameter Kirschner wire was then inserted into the pin tract of the RP model before polymethylmethacrylate was used to construct the patient-specific biocompatible drill template. After removal of the anterior soft tissue, a 2-mm-diameter Kirschner wire was inserted into the cervical pedicle with the assistance of drill template. Cadaveric cervical spines with pin tracts were subsequently scanned using the same CT scanner. A 3D reconstruction was performed of the scanned spines to get 3D models of the vertebrae containing the actual pin tracts. The deviations were calculated between 3D models with virtual and actual pin tracts at the middle point of the cervical pedicle. 3D models of 3.5 mm-diameter screws were used in simulated insertion to grade the screw positions.

**Findings:**

The patient-specific biocompatible drill template was constructed to assist ATPS insertion successfully. There were no significant differences between medial/lateral deviations (*P = *0.797) or between superior/inferior deviations (*P = *0.741). The absolute deviation values were 0.82±0.75 mm and 1.10±0.96 mm in axial and sagittal planes, respectively. In the simulated insertion, the screws in non-critical position were 44/48 (91.7%).

**Conclusions:**

The patient-specific drill template is biocompatible, easy-to-apply and accurate in assisting ATPS insertion. Its clinical applications should be further researched.

## Introduction

Surgical interventions are common for degenerative cervical spine diseases [1], [2], [3]. Due to the three-column fixation property, transpedicular screw fixation via the posterior approach has been shown to have superior stabilization capabilities in several biomechanical and clinical surveys [4], [5], [6], [7]. However, due to the posterior musculature stripping, the posterior approach can cause significant myofascial pain and lead to significant postoperative axial symptoms and neck pain [8], [9], [10]. On the contrary, the anterior approach is less traumatic with no damage to the paravertebral muscles and allows for anterior instrumentation as far as T1 [11], [12]. However, since the screws in the anterior approach are anchored in the cancellous vertebral body, the biomechanical stability of anterior plate fixation is limited, leading to significant failure rates [13]. Thus, for a successful ≥2-level corpectomy or operation for severe traumatic three-column instabilities, a combined anterior and posterior approach was found to be desirable [14], [15], [16] but it would require a secondary posterior approach procedure that might lead to a significant increase in morbidity.

Anterior transpedicular screw (ATPS) in clinical application was first reported by Aramoni et al. [17]. After corpectomy at one to three levels in 9 patients, Aramoni et al. placed ATPS under visualization of the pedicles to affix fibular grafts to cervical pedicles [17]. Koller et al. demonstrated the anatomical feasibility of ATPS and found the pull-out strength of ATPS to be 2.5-fold that of vertebral body screws [18], [19]. The ATPS technique merges the biomechanical merits of posterior transpedicular fixation with the surgical benefits of anterior-approach-only procedures [19], because it can increase initial construct stability in an anterior surgery which is believed to be best beneficial for some severe multilevel cervical instabilities [20], [21], [22].

Accurate insertion is a key to successful application of ATPS in clinic. In fluoroscopy-guided manual insertion of ATPS, a percentage of 78.3% was reported for correctly placed screws and non-critical pedicle breaches in the axial plane [18]. Yukawa et al. reported successful insertion of anterior pedicle screws in 6 patients with the aid of fluoroscopic images of the pedicle axis [23]. However, the sample number was small and the surgery required much time and experienced physicians. Koller et al., using the electronic conductivity device (ECD), found a high accuracy rate of ATPS insertion with no critical screw positioned in the axial or sagittal plane [24]. However, the physical property of ECD prevents it from penetrating the dense cortical bone. Patient-specific drill templates were developed to assist screw insertion using 3D reconstruction, computer aided design (CAD) and rapid prototyping (RP) techniques and good accuracy of screw insertion was obtained [25], [26], [27], [28], [29], [30]. However, these techniques have been used just in the posterior pedicle approach but not in the anterior pedicle approach. Also, the materials used in them, such as photosensitive resin, possess significant cytotoxicity [31], [32]. In the process of surgical drilling, debris of the drill template can get in contact with the wounded area, a potential danger if the debris cannot be totally rinsed.

Therefore, accurate and biocompatible insertion of ATPS remains a challenge. To address this challenge, we first adopted a new strategy to construct a biocompatible drill template for ATPS insertion using 3D reconstruction, RP production and reverse mold manufacture techniques. Secondly, we evaluated the accuracy of the drill template in assisting ATPS insertion.

## Materials and Methods

### Ethics Statement

Ethical approval was obtained from the Human Research Ethics Committee, Southern Medical University, Guangzhou, China. The subjects gave informed consent. And all consent was written in nature regarding body donation for research.

### Specimens

Twenty four formalin-preserved cervical vertebrae (range C2–C7) from four human cadavers (3 males and 1 female, from 52 to 68 years of age, mean 61.5 years) were obtained. The entire specimens were imaged using a Brilliance CT 64-channel scanner (Philips, Eindhoven, The Netherlands). In-plane pixel size was 0.5 mm and slice thickness was 0.705 mm. All the 48 pedicles in the 24 cervical vertebrae were used after the CT scan images showed no significant bone defects.

### Three-dimensional Reconstruction of Cervical Models with Virtual Pin Tracts

**Figure 1:**
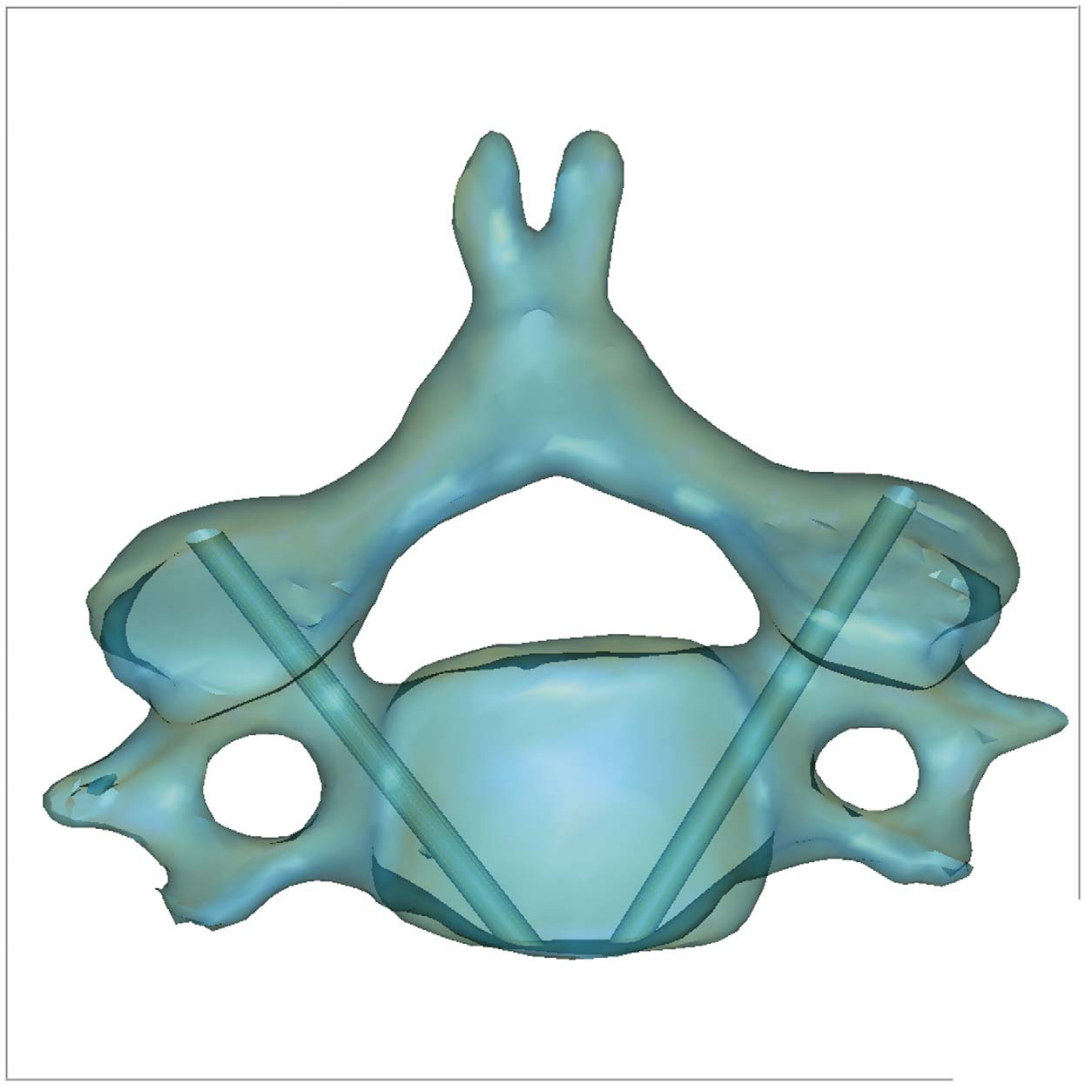
3D model of cervical vertebra with virtual pin tracts. 3D model of each vertebra was reconstructed in Mimics software. The 2 cylinders with 2 mm-diameters were then imported and their locations were ensured at the central cervical pedicle by visual observation. With the tool “subtraction” under “Boolean operation” in the Geomagic studio® software, the 3D model of the cervical vertebrae with bilateral pin tracts was obtained and saved in group 1 in (.stl) file format.

The datasets of cervical specimens were processed and edited with Mimics software v14.11 (Materialise Corp., Leuven, Belgium). 3D reconstructions were obtained from the 2D CT images. An interactive image processing strategy, such as “Threshold” and “Region growth”, was used to segment the contours of each vertebra to obtain the 3D-reconstructed models. The 2 cylinders, 2 mm in diameter and pre-designed in Unigraphics NX 6.0 (Siemens PLM Software, Plano, TX), were imported into Mimics software where it could be freely translated and rotated. It was made sure that the cylinders were located at the central cervical pedicle by visual observation. With the tool “subtraction” under “Boolean operation” in the Geomagic studio® software, 3D models of cervical vertebrae with bilateral pin tracts were obtained (Figure 1) and saved in group 1 in stereolithography file format (.stl) supported by many software packages and widely used for rapid prototyping and computer-aided manufacturing.

### Production of Biocompatible Drill Templates

**Figure 2:**
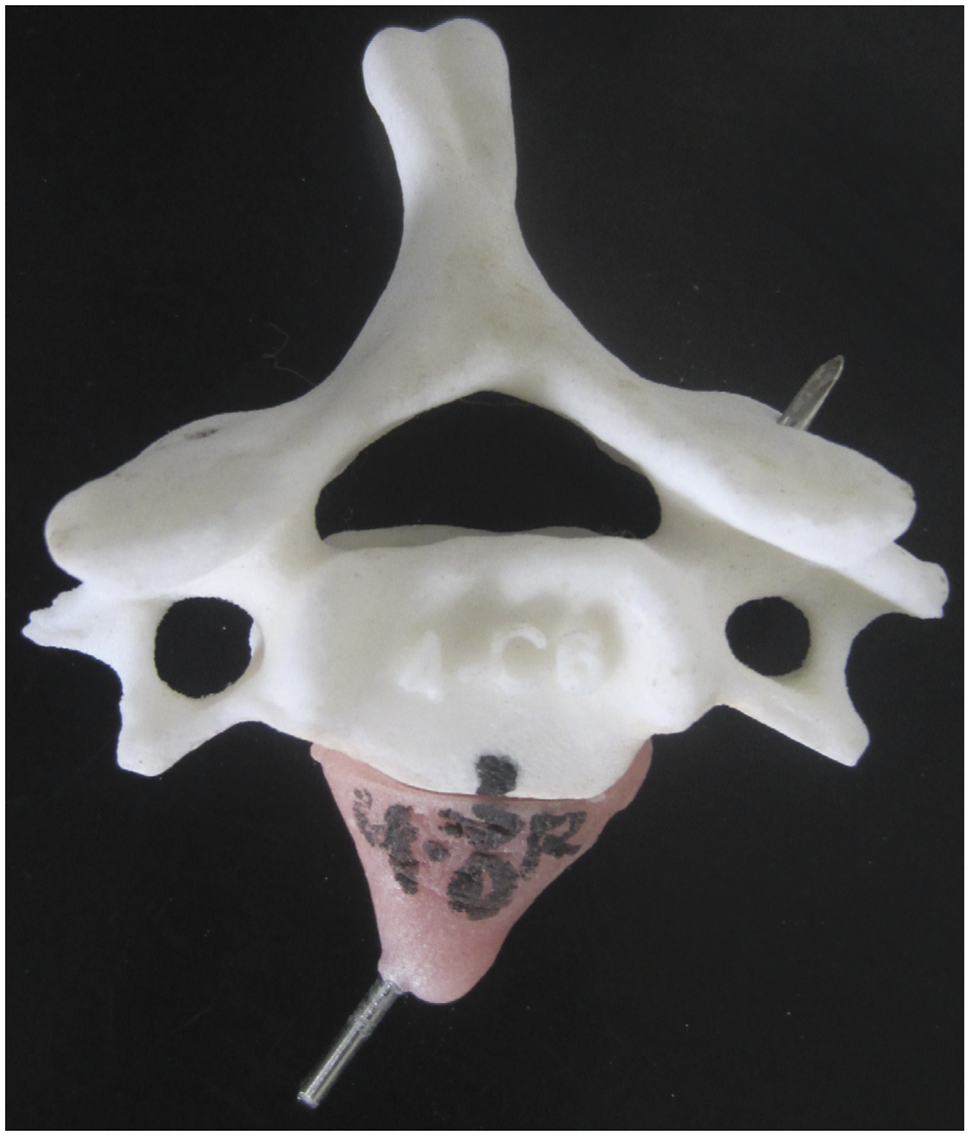
Production of biocompatible navigation template with RP model. The 3D model of cervical vertebrae with virtual pin tracts was rapid-prototyped with Z Corporation 3D printer Spectrum Z™510. A Kirschner wire was then inserted into the pin tract of the RP model and polymethylmethacrylate (PMMA) was used to construct the drill template.

The “.stl” files of cervical vertebrae were processed using the software Zprint 7.10 (Z Corporation, Burlington, MA) and printed on the Z Corporation 3D printer Spectrum Z™510 (Z Corporation, Burlington, MA). The 3D models were virtually cut into thin layers of 0.0875 mm intervals with the Zprint 7.10 software and transferred into the Spectrum Z510 for rapid prototyping (Figure 2). A Kirschner wire was then inserted into the pin tract of the RP model. Polymethylmethacrylate (PMMA), which is usually used as bone cement and has good biocompatibility [33], [34], [35], was used to construct the drill templates (Figure 2). Also, to allow for convenience and easy handling, a grip was created at the top of the drill template. In addition, since the surgery field was narrow, the base of the drill template was not allowed to exceed the juncture of the vertebral body and the transverse process. After the PMMA was solidified, the Kirschner wire was pulled out to finalize the drill template.

### Cadaveric Kirschner Wire Insertion

**Figure 3:**
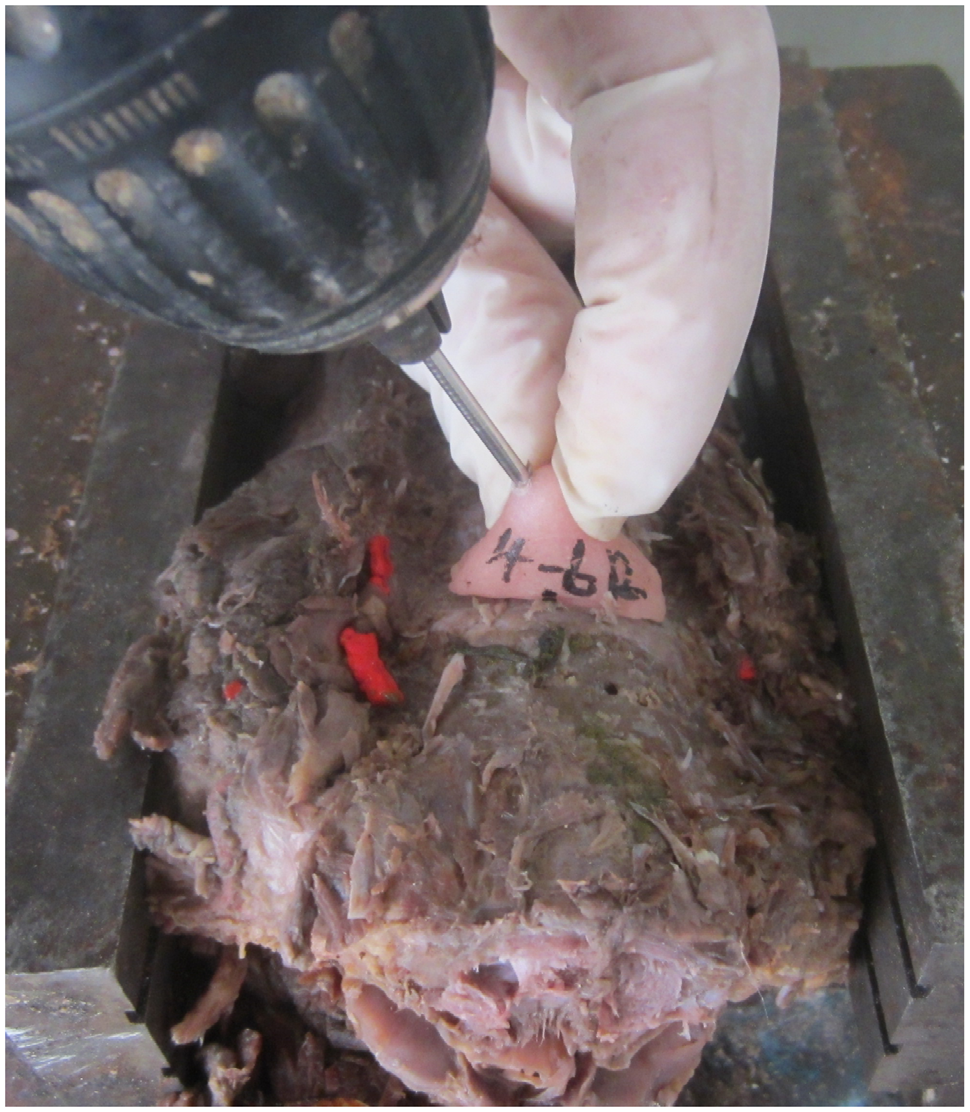
Actual drill with navigation template in cadaveric cervical specimens. Anterior soft tissue was removed from the vertebrae. The drill template was put in place by hand and compressed slightly to the anterior surface of cervical vertebrae. A 2 mm-diameter Kirschner wire was then drilled into the cervical pedicle with the assistance of the drill template.

Anterior soft tissue was removed from the vertebrae. The drill template was put in place by hand and compressed slightly to the anterior surface of cervical vertebrae. A 2-mm-diameter Kirschner wire was then drilled into the cervical pedicle with the assistance of the drill template (Figure 3).

### Secondary 3D Reconstruction of Cervical Models with Pin Tracts

After all pin tracts were drilled, the cadaveric cervical specimens were scanned with the same CT scanner using the same parameters. Since the Kirschner wires produced image artifacts, they were pulled out before the image acquisition. 3D models of each vertebra with pin tracts were obtained with same segmentation and reconstruction strategy, and saved in group 2 in “.stl” file format.

### Assessment of Accuracy of Screw Insertion

Accuracy of ATPS insertion with the assistance of the drill templates was evaluated by a reverse engineering process using the software Geomagic studio®, version 11 (Geomagic, Inc., Morrisville, NC). The 3D models of groups 1 and 2 were imported into the Geomagic software, and the deviations at the middle point of the pedicle in the axial and sagittal planes were calculated. The axial plane’s deviations towards the lateral side were recorded as positive values and the deviations towards the medial side as negative values. The sagittal plane’s deviations towards the superior and inferior sides were recorded as positive and negative values, respectively.

**Figure 4:**
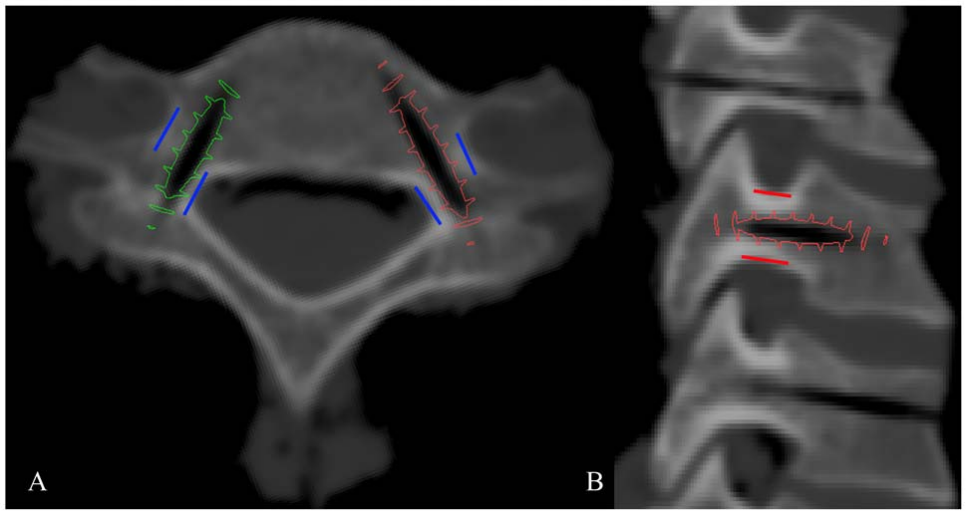
Accuracy evaluation with screw simulation insertion in the axial (A) and sagittal (B) planes. The blue lines in Figure 4A are the border of cervical pedicle in axial plane. The structures inside and outside the blue lines are vertebral canal and vertebral artery, respectively. And the red lines in Figure 4B are the border of cervical pedicle in sagittal plane. The structures upper and lower the red lines are foramen intervertebrale. A pre-designed 3D screw model (3.5 mm in diameter), which aligned with the pin tract of the 3D model of group2, was imported into Mimics to simulate the screw insertion. The screw positions were graded according to the distance between the screw thread and the border of pedicle cortex.

**Figure 5:**
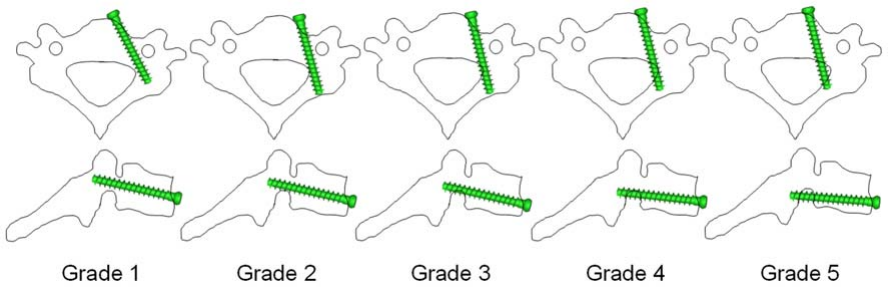
Illustration of grades of ATPS positions in cervical pedicle. Grade 1: Screw positioned at the center of the pedicle. Grade 2: Less than one-third of the screw cross-section (≤1.2 mm with a 3.5-mm diameter screw) penetrating the cortex. Grade 3: Between one-third and one-half of the screw cross-section penetrating the cortex (or deviation <2 mm). Grade 4: More than one-half of the screw cross-section penetrating the cortex (or deviation ≥2 mm). Grade 5: Deviation equal or greater than the screw diameter.

Aligned with the pin tract of the 3D model of group 2, a pre-designed 3D screw model (3.5 mm in diameter) was imported into Mimics to simulate the screw insertion (Figure 4). A grading was used to distinguish non-critical and critical screw positions [18], [24], [36]. Briefly, the grading consists of the following (Figure 5):

Grade 1: Screw positioned at the center of the pedicle.

Grade 2: Less than one-third of the screw cross-section (≤1.2 mm with a 3.5-mm diameter screw) penetrating the cortex.

Grade 3: Between one-third and one-half of the screw cross-section penetrating the cortex (or deviation <2 mm).

Grade 4: More than one-half of the screw cross-section penetrating the cortex (or deviation ≥2 mm).

Grade 5: Deviation equal or greater than the screw diameter.

Non-critical pedicle breaches corresponded to grades 1 and 2. Critical pedicle breaches, with the potential of posing a risk to the vertebral artery (VA), nerve root or dural sac, corresponded to grades 3–5 [24].

### Statistical Analysis

Independent-sample T test was used to analyze the screw direction differences between the deviations towards lateral and medial in the axial plane and towards superior and inferior in the sagittal plane. A *P* value <0.05 was considered as statistically significant. And, to show the real deviations, the absolute deviation values were calculated to get their means and standard deviations in the axial and sagittal planes, respectively.

With the scoring system [18], [24], assessment of pedicle screw position could be performed both in the axial and sagittal plane. Each point was assigned to each of the five grades of screw position. The accuracy score in the axial plane (range 1–5 points) and the accuracy score in the sagittal plane (range 1–5 points) was summed and described as the accuracy sum score with its maximum being 10 points and the minimum being 2, delineating most accurate screw placement.

## Results

With the 3D reconstruction, rapid prototyping production and mold manufacture techniques, the patient-specific biocompatible drill templates were constructed successfully. During the operation, the drill template fit the position easily to allow no significant free motion between the drill template and the anterior cervical surface. The Kirschner wires were inserted into the cervical pedicle easily with the assistance of the patient-specific biocompatible drill template.

**Figure 6:**
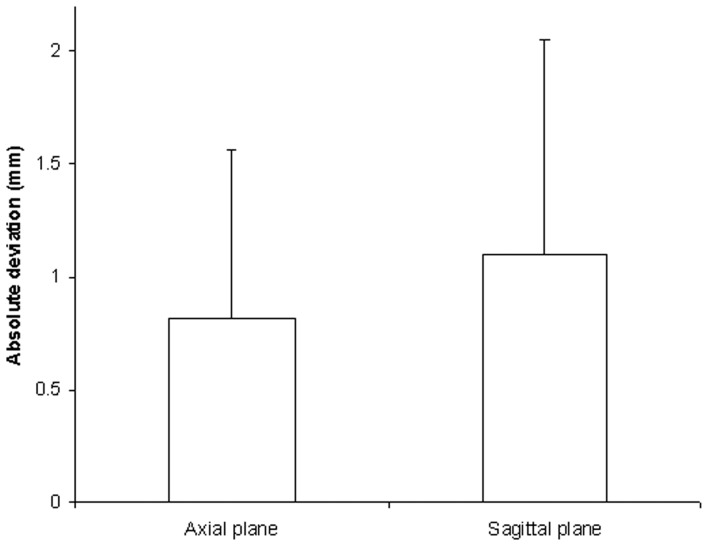
Absolute deviation values in the axial and sagittal planes.

Calculation showed no significant difference between the deviations towards lateral and medial in the axial plane (t = −0.258, *P = *0.797). The absolute deviation value in axial plane was 0.82±0.75 mm (Figure 6). There was no significant difference either between the deviations towards superior and inferior in the sagittal plane (t = 0.332, *P = *0.741). The absolute deviation value in the sagittal plane was 1.10±0.96 mm (Figure 6).

In the simulated insertion of 3.5 mm-diameter screw, one screw position was grade 3 (2.1%), one was grade 2 (2.1%) and the others were grade 1 (95.8%) in the axial plane. The mean accuracy score in the axial plane was 1.02. Three pedicle screw positions were grade 3 (6.25%), 4 were grade 2 (8.3%) and the others were grade 1 (85.4%) in the sagittal plane. The mean accuracy score in the sagittal plane was 1.22. The accuracy sum score showed a mean of 2.27 points with a possible total of 10 points. Summing up the screw positions in the axial and sagittal planes, the screws in a non-critical position were 44/48 (91.7%) and those in a critical position were 4/48 (8.3%).

## Discussion

Pedicular screw insertion has generally been considered to be very risky because it can injure VA, the spinal cord or nerve roots seriously. Because of few landmarks on the anterior surface of cervical vertebra and also a relative long distance between the anterior surface and the pedicle, insertion of ATPS is more difficult and dangerous than posterior transpedicular fixation. Several methods have been explored for precise anterior cervical transpedicular screw placement, including the fluoroscopy-guided manual insertion [18], [19], fluoroscopic images of the pedicle axis [36] and ECD [24]. It is also possible to use CT-based and fluoroscopy-based computer assisted surgeries (CAS) to assist insertion of ATPS because they have been used to assist screw insertion in posterior transpedicular fixation and yielded high accuracy [37], [38], [39]. The rate of pedicle perforation in posterior cervical or cervical-thoracic fixation using pedicle screws was 8.6% in the conventional group and 3.0% in the CAS group [37]. Gelalis et al. [38] found the percentages of screws fully contained in the pedicle ranged from 89 to 100% using CT-based CAS and 81 to 92% using fluoroscopy-based CAS. However, several caveats should be considered: (1) the learning curve to master these complex techniques is relatively long; (2) errors may occur when adjacent segments of the spine shift intraoperatively or if the registration frame and optical array shift; (3) tracking of optical array devices can be obscured by the surgeon or surgical tools; (4) cost of equipment is high; and (5) surgical time is long [25].

The patient-specific drill templates eliminated the need for expensive equipment and a time-consuming procedure in an operating room [26]. They were initially used in hip and knee surgery [40] and so far have been developed to assist screw insertion in cervical surgery, yielding good accuracy of screw insertion [25], [26], [41], [42], [43]. In the present study we further improved the non-biocompatible drill templates into biocompatible ones. We first produced a non-biocompatible RP model of cervical vertebra with pin tracts. After inserting the Kirschner wire, we used PMMA, a biocompatible material [33], [34], [35], to produce the reverse mold of the RP model and construct a biocompatible drill template. Moreover, we improved the complex design of previous patient-specific drill templates which is beyond the ability of a surgeon because it requires not only medical knowledge but also knowledge of reverse engineering and CAD design [25]. First the drill template we constructed is easy to produce. It does not need surgeons to design the reverse surface modeling with complicated CAD technology. Since every step is programmed in certain software, an operator can just produce it step by step. Secondly, our drill template is easy to use. During a surgery, our drill template can find its position easily and fit the anterior cervical surface so well that the entry point and direction can be accurately determined. Next the Kirschner wire can be drilled into the cervical pedicle. Consequently it is possible for a surgeon to design customized surgical plan preoperatively. The technique we used can thus eliminate the need for complex equipment and markedly reduce the long duration of the surgery.

To ensure the accuracy of our drill template, we calculated the deviations at the central point of the cervical pedicles to get quantitative data. In this way we could evaluate the accuracy more directly. In the present study, we also calculated the absolute value of the deviations to show the real deviations because the deviations at different directions had positive and negative values, which resulted in a significantly smaller mean. Our study showed that the absolute value deviations in the axial plane (0.82±0.75 mm) and in the sagittal plane (1.10±0.96 mm) might be within an acceptable range in clinical application.

Gelalis et al. [38] found that the screws positioned with free-hand technique tended to perforate the cortex medially whereas the screws placed with CT navigation guidance seemed to perforate laterally more often. However, in our study, we found no significant differences between the medial and lateral deviations in the axial plane or between the superior and inferior deviations. Our finding means that there is no specific direction guidance resulting from our biocompatible drill templates.

The simulated insertion of 3.5 mm-diameter screw can show the real implant-pedicle anchorage. Our grade results are very close to the ECD results [24]. Unfortunately, we had 4 screw positions in grade 3. This means the screws penetrated the pedicle cortex from 1/3 to ½ of the screw cross-section. The deviations are relatively large although it is reported that surgeons judged the pedicle screw position of grade 3 as ‘indeterminate’ or ‘borderline’ because it would not cause injury to the VA or nerve root but rather would push the either one away [36]. We also found the virtual pin tracts had some deviations from the midline of the pedicle due to the error from our visual observation. This is one of the limitations of our research. In further research, optimal feature of the pin tract should be extracted from the irregular morphology of the cervical pedicle to make sure the pin tract at the midline of the cervical pedicle [25], [26]. Another limitation of ours is that the shape of drill templates was not optimized for clinical application. This is a primary study of ours on the biocompatible drill template. Future shape optimizing will follow the easy-to-apply and artistic principles. And pin tracts will be extended as long as possible to further improve the accuracy in assisting ATPS insertion.

### Conclusion

In this in vitro study, the patient-specific drill template we constructed is compatible, easy-to-apply and accurate. Further research should be done to test its clinical applications.
